# Advances in the One-Step Approach of Polymeric Materials Using Enzymatic Techniques

**DOI:** 10.3390/polym15030703

**Published:** 2023-01-30

**Authors:** Richard Ansah Herman, Xuan Zhu, Ellen Ayepa, Shuai You, Jun Wang

**Affiliations:** 1 School of Materials Science and Engineering, Jiangsu University of Science and Technology, Zhenjiang 212100, China; 2 Jiangsu Key Laboratory of Sericultural Biology and Biotechnology, School of Biotechnology, Jiangsu University of Science and Technology, Zhenjiang 212100, China; 3 Oil Palm Research Institute, Council for Scientific and Industrial Research, Kade P.O. Box 74, Ghana; 4 Key Laboratory of Silkworm and Mulberry Genetic Improvement, Ministry of Agricultural and Rural Affairs, Sericulture Research Institute, Chinese Academy of Agricultural Sciences, Zhenjiang 212100, China

**Keywords:** one-step synthesis, enzymatic approach, polymer materials, application

## Abstract

The formulation in which biochemical enzymes are administered in polymer science plays a key role in retaining their catalytic activity. The one-step synthesis of polymers with highly sequence-controlled enzymes is a strategy employed to provide enzymes with higher catalytic activity and thermostability in material sustainability. Enzyme-catalyzed chain growth polymerization reactions using activated monomers, protein–polymer complexation techniques, covalent and non-covalent interaction, and electrostatic interactions can provide means to develop formulations that maintain the stability of the enzyme during complex material processes. Multifarious applications of catalytic enzymes are usually attributed to their efficiency, pH, and temperature, thus, progressing with a critical structure-controlled synthesis of polymer materials. Due to the obvious economics of manufacturing and environmental sustainability, the green synthesis of enzyme-catalyzed materials has attracted significant interest. Several enzymes from microorganisms and plants via enzyme-mediated material synthesis have provided a viable alternative for the appropriate synthesis of polymers, effectively utilizing the one-step approach. This review analyzes more and deeper strategies and material technologies widely used in multi-enzyme cascade platforms for engineering polymer materials, as well as their potential industrial applications, to provide an update on current trends and gaps in the one-step synthesis of materials using catalytic enzymes.

## 1. Introduction

Biocatalysis, which is broadly defined as the utilization of enzymes to catalyze chemical reactions, is utilized in numerous fields of application. Since the beginning of the twenty-first century, enzymes have been gaining popularity and a wide range of biocatalysts are employed in an increasing number of processes operating at up to an industrial scale, particularly in the field of synthesizing polymeric materials and synthetic chemistry [1]. Synthetic chemists are beginning to appreciate the significance of enzymes associated with the extreme selectivity of these natural (or engineered) catalysts, along with the benefits of using them under environmentally acceptable reaction conditions. Materials’ service performances in a variety of disciplines, including biomedical devices [2], energy storage accumulators [3], and environmental cleanup systems [4], are greatly influenced by the synthesis of the technologies applied. Significant work has been put into creating various modification techniques to finely adjust the surface structures and properties of materials [5,6], endowing those materials with abundant functions and properties. Conventional modification techniques, on the other hand, frequently suffer from difficult chemical or physical processes, high solvent or energy consumption, or required preparation for the substrate materials [7], hence the need to develop means that produce sustainable synergy between the material and its practical properties. Practically, a desirable modification strategy should include the following characteristics: natural origin, environmentally benign procedure, mild reaction condition, strong binding forces to the substrate surface, simplicity of implementation, and adaptable functionalization accessibility.

Enzyme-catalyzed polymer synthesis has received a lot of attention lately due to its possible usage in fuel cells and organic synthesis. The implementation of far more complex synthesis schemes is made possible using multiple enzymes. By using or manipulating the metabolic networks of biological systems for catalysis, one-step synthesis can be carried out in those systems. Enzyme use in chemistry has a long history, including applications in the textile, food and detergent industries [8]. Likewise, the advent of on-demand enzyme production, manipulation, and modification contributed to the development of current biocatalysis [9,10]. As an alternative, isolated biocatalysts can be used to perform multi-enzymatic catalysis in vitro. Both methods, in vivo and in vitro, offer unique benefits, drawbacks, and difficulties that will be demonstrated using current instances. The current method is very helpful in modifying the polymeric structures of materials that would otherwise be impossible via chemical synthesis due to the mild circumstances in which the enzyme-catalyzed process is carried out. Additionally, multi-enzymatic tandem processes that power life exhibit unparalleled catalytic efficiencies due to biological reactor characteristics such as compartmentalization, nano-confinement, and out-of-equilibrium dynamics [11].

In addition to the tremendous incentive to develop chemical methods that are more ecologically friendly, faster access to proteins and the ability to alter them significantly influenced the shift in industrial thought [12,13]. Enzymes are capable of competing with traditional chemical (catalytic) processes for a variety of chemical changes, even ones that were previously regarded as unnatural. An excellent illustration is the discovery of the catalytic activity of aldoxime dehydratases’ in eliminating the Kemp process of the deprotonation of a benzisoxazole ring in base catalysis, for which, except from chemical bases, only recently developed proteins were recognized to be efficient catalysts [14]. Enantioselective methods using hydrolases made it possible to employ enzymes in the synthesis of asymmetric polymers, particularly when biocatalytic (dynamic) kinetic resolutions were involved. The main enzymatic techniques that are widely applicable in the one-step synthesis of polymeric materials are discussed in this review, with an emphasis on the reactions created in the last decade.

## 2. One-Step General Approach

**Figure 1:**
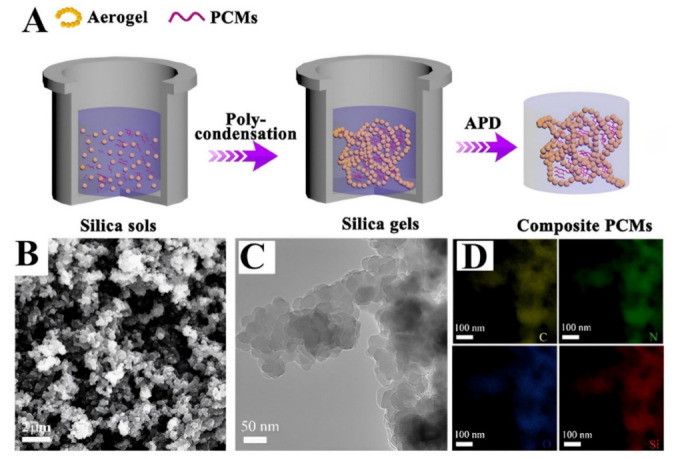
Phase change materials to improve thermal conductivity prepared in a one-step synthesis. (**A**) Illustration of the silica aerogel-based composite PCMs’ schematic synthesis, (**B**) a scanning electron microscope image of the silica aerogel, and (**C**) a transmission electron microscope image, consisting of primary nanoparticles (approximately 30 nm) and aggregated nanoparticles in a linked, three-dimensional porous structure. (**D**) TEM mapping images of the silica aerogel consisting of evenly distributed C, N, O, and Si elements [22], with permission from Elsevier, copyright (2020).

The one-step approach refers to the process that produces the desired product by running a single reaction, especially where there are alternative pathways to the same product involving two or more reactions in a sequence. The excellent chemo, regio-, and stereoselectivity of enzymes is the driving force behind the interest in employing them in one-step synthesis. Enzymatic techniques used in polymeric reactions are underpinned by ideas comparable to those used in organic synthesis. The one-step technique has recently emerged as a key technology for the synthesis of stable, affordable, and environmentally friendly supported materials or composites [15,16]. According to Liu et al. [17,18] who employed the one-step synthesis in 3D NaV_2_O_5_ mesocrystal as mode materials, the advantages of the method are that it can control the synthesis conditions of the dual metal sulfide composites, simplify the experimental process, and shorten the reaction time. This technique can avoid the problematic instability of a particular polymer substance or other intermediates, as well as skip the tedious purification processes. Comparing the one-step general approach to a multi-step method, the one-step method is facile and secured, controlled material size, fast, easy and efficient synthesis, while the multi-step synthesis may require long chains, and complicated methods leading to low product yield as well as large energy utilization. The development of novel processing and synthetic processes has made it possible to combine several preparation procedures into the one-step approach. To develop consistent metal–silica–polymer core-shell–shell templates for metal-carbon yolk-shell nanostructures, Priestley et al. developed a simple one-step Stöber technique [19,20]. The restricted volume one-step synthesis of silver-decorated polymer colloids with antibacterial and sensing characteristics was reported. The sensitivity of the colloids to hydrogen peroxide reveals a linear response across a wide concentration range and possesses strong antibacterial characteristics [21]. Recently, numerous investigations have been conducted to improve the thermal conductivity of phase change materials (PCMs) [22]. These PCMs are potential alternatives for thermal energy storage because they may selectively absorb and emit enormous amounts of heat during the process of phase shifting [23,24,25,26]. Figure 1 indicates that while PEG or octadecanol acts as the PCM, silica aerogel acts as a supportive substance to prevent PCMs from leaking during the phase transition process. The porous channels of the 3D silica aerogel are specifically occupied by the hydrolytic polycondensation of bridging silsesquioxane precursor and organic PCMs in situ, which makes the production of silica aerogel easier. Finally, without additional solvent exchange or surface modification, the composite PCMs are made utilizing the APD method. The in-situ one-step preparation of a monolithic silica aerogel-based composite phase change materials for thermal protection was constructed. Due to the synergistic combination of the monolithic silica aerogel-based composite PCMs’ high latent heat capacity and poor thermal conductivity, the period of heat preservation might be prolonged. This material has the potential to be directly applied to thermal protection and insulation devices [22].

Novel materials are synthesized using end-functionalized hydrogenated polymers derived from nitrile-butadiene rubber (NBR), and are well suited for use as adhesives and sealing materials in a variety of applications [27]. According to Liu et al. [28], the one-step synthesis of end-functionalized hydrogenated nitrile-butadiene rubber by combining the functional metathesis with hydrogenation has an advantage of short synthesis path, excellent yield, and complies with the regulations of green chemistry. Due to its mild conditions, environmental tolerance, high efficiency, and adjustable performance, the olefin metathesis reaction has attracted substantial attention recently for the synthesis of rubber [29,30]. High internal phase emulsions (HIPEs), double emulsions, and other complex emulsions make excellent templates for the development of porous polymeric materials. The surfactant-free emulsions with erasable triggered phase inversions conducted via a one-step approach revealed that the polymer can be employed repeatedly for porous materials and be tunable for complicated emulsions because CO_2_ triggering is erasable [31]. The one-step process makes creating porous 3D scaffolds and particles out of the block copolymer very convenient. Based on a one-step approach for lipase immobilization, Lu et al. synthesized SiO_2_-coated Fe_3_O_4_ nanoparticle/polyacrylonitrile beads to immobilize *C. antartica* lipase B, with an immobilization yield of up to 98.4% [32].

Specifically, the one-step synthesis of polymeric materials has been conducted in the hydrothermal synthesis of carbon dot–polymer composites [33], SnO_2_ electrodes by UV curing technology [34], photoluminescent core/shell polystyrene particles [35], the radiation of gel polymer electrolytes [36], magnetic chitosan polymer composite films [37], nitrogen and sulfur–codoped graphene electrodes [38], vegetable oil-based acrylate prepolymers [39], the biodegradation and biocompatibility of polyesters [40], transparent high refractive index sulfur-containing polymers [41], the synthesis of Mn_3_O_4_ on carbon cloth [42], and recyclable crosslinked polymer networks [43]. The one-step reaction is the preferred approach for modifying polymer materials due of its advantages of ease of use and mild conditions. Additionally, a one-step synthesis of a foundational material also greatly reduces cost and improves prospective economic feasibility from the perspective of product development.

## 3. Enzymes Catalyzed One-Step Synthesis

### 3.1. Free Enzymes

**Figure 2:**
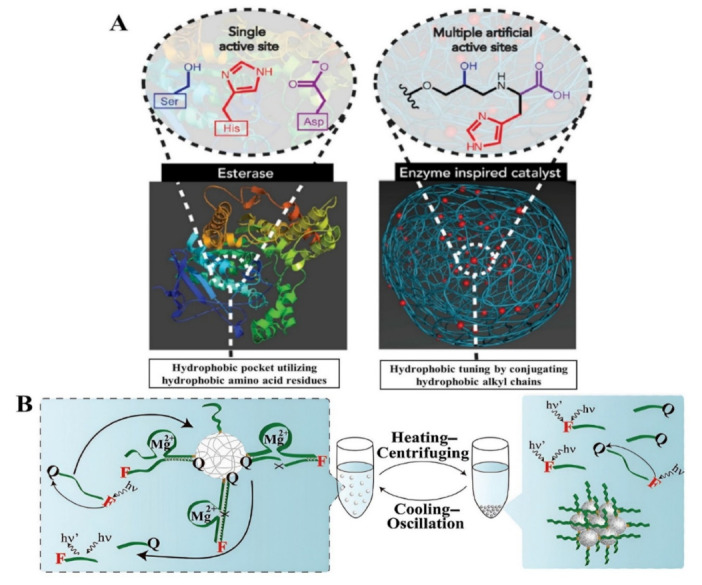
(**A**) Typical enzyme-catalyzed reactions, comparing the natural catalytic active site and triad of esterases and the artificial enzyme mimic integrated into the polystyrene Merrifield resin, demonstrating various catalytic sites and hydrophobic tailoring enabled by functionalized resins [51], with permission from Elsevier, copyright (2017). (**B**) A representation of the heating–centrifuging and cooling–oscillation cycle for the recycling of pNIPAM/DNAzyme microgels [46] with permission from Wiley periodicals Inc. (2022).

Enzyme-catalyzed reactions can be carried out in gentle conditions with respect to temperature, pressure, and pH as well as in solvents that are more environmentally friendly (such as water) or in mixed solvent systems (Figure 2A). Enzymatic approaches have been substantial in their applications as catalysts for polymeric one-step synthesis. A facile enzymatic one-step preparation of fluorescent conjugated polymers of phenols using horseradish peroxidase (HRP) as the catalyst resulted in the total polyphenols exhibiting fluorescence signals with significant shifts rendering them valuable for use in fluorescence quenching-based sensors [44]. Additionally, surface modified electrospun microfibers as suitable supports for protein immobilization using HRP was reported [45]. The one-step precipitation polymerization of high catalytic and recyclable DNAzyme was used to develop complex catalytic functionalized poly-*N*-isopropylacrylamide (pNIPAM) microgels [46] (Figure 2B). A recyclable bi-enzyme system for glucose sensing was formed by the Mg^2+^-dependent DNAzyme and the hemin-G-quadruplex horseradish peroxidase (HRP)-mimicking DNAzyme, with the microgel catalysts maintaining 80–91% after eight times of recycling [46]. The recovery of α (alpha)-amylase from an *Aspergillus oryzae* culture supernatant in one easy step by creating an insoluble combination with the Eudragit^®^ copolymer provides an efficient method that allows the recycling of the polymer after precipitation [47]. An anionic cellulase was joined with a copolymer poly(ethyleneglycol)-graft-poly (N, N-dimethylaminoethyl methacrylate) (PEG-g-PDMAEMA) with hydrophilic and cationic chains to develop a water-soluble cellulase/PEG-g-PDMAEMA complex. Charged copolymers could be successfully inhibited by turning the cellulase system based on a synergetic polymer pair system on/off and recover positively charged polymers [48]. Additionally, the enzymatic grafting of ferulic acid with cellulose to enhance matrice–fiber compatibility in bio-composites was achieved via one-step synthesis. The resulting bio-composites demonstrated a 12% drop in Young modulus, and a 23% improvement in the elongation under maximum stress, indicating a material with higher mechanical resistance [49]. Multiple oligonucleotide partzymes constitute multicomponent nucleic acid enzymes (MNAzymes), which only associate to develop catalytic complexes when a target nucleic acid is present. According to Hanpanich et al. [50], LNA-modified MNAzymes aided by cationic copolymer for the one-step isothermal RNA detection improved the robustness of the MNAzyme which could pave the way for the design of an alternative rapid, accurate, isothermal, and a protein-free RNA diagnostic tool, which is anticipated to have significant therapeutic implications. Table 1 shows the overview of some free and immobilized enzymes employed in the generation of polymer materials via the one-step approach.

### 3.2. Immobilized Enzymes

The immobilization of enzyme polymer synthesis on or within a support is crucial for both scientific and commercial purposes [74,75,76,77,78]. Most conventional immobilization techniques involve the chemical grafting, embedding, and adsorption of enzymes onto organic or inorganic carriers [79]. Magnetic nanoparticles [80], mesoporous silica [81], and organometallic skeletons [82] are a few immobilized carriers that have been reported. They have the potential to be used in numerous sustainable applications due to their reusability, simple operation, strong regio-selectivity, and moderate reaction conditions [83,84,85]. A stimulus responsive to control enzyme activity for one-step synthesis was designed, and the use of immobilized enzymes in stimulus-sensitive hydrogels, hydrogel particles, or hydrogel coatings was regarded as another common strategy [86]. It was reported that the degree of gel swelling can be changed to affect enzyme activity, and the following factors are most likely to contribute to the changes in enzyme activity upon deswelling: altered substrate accessibility to the active site; altered enzyme conformation and moving flexibility as a result of packing the enzyme being packed within the hydrogel meshes; changed enzyme environment (hydrophilicity, presence of charged groups); and modified substrate/product diffusion due to variations in water content [86]. Küchler et al. [87] immobilized an enzyme on silicate glass through simple adsorption of dendronized polymer–enzyme conjugates by employing a one-step synthesis technique and concluded that the methodology could be considered as a promising platform for localizing active enzymes on solid supports. Recently, insoluble substrates for bioconversion efficiency have been frequently low due to the enzyme’s restricted access to the insoluble substrate when it is mounted on insoluble supports. Hence, it is possible to think of stimuli-responsive polymers as carrier materials as a workable solution to the aforementioned issues via one-step synthesis. Polymers that respond to stimuli can react to changes in the environment fast and correctly. According to their reactive situations, stimuli-responsive polymers may be classified as redox-responsive polymers, temperature-sensitive polymers, photo-responsive polymers, and pH-responsive polymers [88,89]. Huang et al. [89] reported that one-step immobilization of β-glucosidase by recyclable UCST-responsive polymers are a potential agent for green biocatalysis. There is strong synthetic usefulness and economic interest in polymers having end functional groups, such as macromonomers and telehelic end functionalized macromolecules. They can be synthesized using a variety of techniques such as chemically modifying the ends of their polymer chains and polymerizations. *Candida antartica* lipase B immobilized for the one-step lipase-catalyzed production of α,ϖ-thiophene-capped poly(-caprolactone) macromonomers is potentially very significant to produce ɛ-CL based polymers with various end functionalities [90]. Thus, enzyme immobilization from free enzymes and on polymer surfaces as carrier materials could maximize the one-step polymerization and synthesis of polymeric materials for efficient scalable laboratory, industrial, and environmental utilization.

## 4. Cascade Reaction Systems in One-Step Synthesis

A biocatalytic “cascade” or “domino” reaction is what is referred to when an enzyme-catalyzed first step results in the development of an unstable intermediate which then spontaneously performs additional reactions to generate the stable product. Therefore, we would want to define a biocatalytic cascade as any reaction system that uses at least one biocatalyst and involves two or more simultaneous transformations occurring in the same reaction vessel. This includes enzyme-initiated spontaneous sequences, chemo-enzymatic sequences, and multi-enzymatic sequences [91].

### 4.1. Multi-Enzyme Cascade Reaction Systems

**Figure 3:**
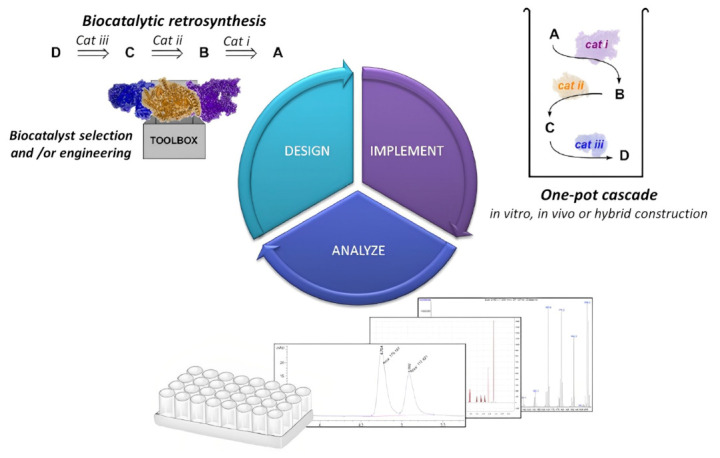
A multi-enzyme cascade development cycle that has been analyzed and implemented. Whereas a wider variety of biocatalysts are now readily available, both in vitro and in vivo de novo enzyme pathway development is becoming easier using a biocatalytic retrosynthetic strategy [103], with permission from the American Chemical Society, copyright (2017).

Multi-enzyme systems of remarkable complexity have been developed and offer considerable advantages including the demand of time, reversible reactions driven to completion, reduced costs and chemicals for product recovery, and minimal exposure to a concentration of unstable compounds. Top-down research on the composition and operation of a multi-enzyme complex has received a lot of attention [92]. Additionally, the bottom-up design of artificial multi-enzyme systems, which entails the selection of enzymes, design of polymer linkers and catalytic link sites, and controllable assembly for desired structure and function, has a considerable measure of work to conduct than natural multi-enzyme complexes, which have evolved over the years [93]. The only disadvantage of a multi-enzyme cascade reaction will be the poor stability and reusability of the enzyme with a tendency to inactivate in toxic media or very high temperatures. Enzymatic cascade reactions, which combine many enzyme reactions in a single vessel without isolating intermediates, hold considerable promise for the development of sustainable chemical processes [94]. Catalyzing two or more enzymes in a cascade system is required for many in vivo biological processes. The results of the previous enzyme catalysis may be used as the substrate for the subsequent enzyme catalytic reaction. Due to the selectivity and high efficiency of enzyme catalysis, complex transformations may be efficiently accomplished by a sequence of enzyme cascade catalytic reactions without any intermediate interruption [95,96,97]. Enzyme catalytic reactions have been applied to the synthesis of polymers, and this has generated considerable interest. Figure 3 shows a biocatalytic one-step retrosynthesis system for multi-enzyme cascade development. To fabricate functional polyamides with a variety of topologies and stimuli-responsive properties, Zhuang et al. [98] developed a cascaded step-growth polymerization. These polymers in the existence of biologically important reductants can degrade molecules through backbone degradation with adjustable kinetics. It has been reported that biocatalysis in polymersomes can be used to compartmentalize multi-enzyme cascades with incompatible reaction steps. The results indicated that reintegrating extremely selective protein channels into the membrane prevents cross-inhibitions in cascade reactions. However, the need to maintain compartmentalization while reducing mass transport restrictions across the polymer membranes is difficult [94,99]. In a biocatalytic one-step cascade synthesis of _L_-tyrosine derivatives using benzenes, NH_3_ and pyruvate as substituted starting materials, crude gasoline blends comprising of toluene were utilized to yield more than 97% of 3-methyl-_L_-tyrosine [100]. Additionally, in a biological cascade reaction, an enzyme cascade system with glucose oxidase (Gox) enclosed in a metal organic framework (ZIF-8) for the oxygen-tolerant RAFT polymerization catalysis of reusable biomimetic mineralization via a one-step facile synthesis highlights the processes in maintaining similar composite activity and comparable to the original composites following five reuses. In this regard, the polymerization process and the first-order kinetic standards are both suitable to influence how the molar mass and conversion relate to each other [101]. Fischereder et al. reported a one-step cascade fashion involving two enzymatic reaction steps performed simultaneously by employing transaminases and strictosidine synthases in the stereoselective cascade to C_3_-methylated derivatives. Depending on the enzymes used in a Pictet–Spengler reaction catalyzed by strictosidine synthases, the selected amines condensed with secologanin produced pure diastereomeric products (>98% excess) [102].

### 4.2. Synthetic Cascade Reaction Systems

**Figure 4:**
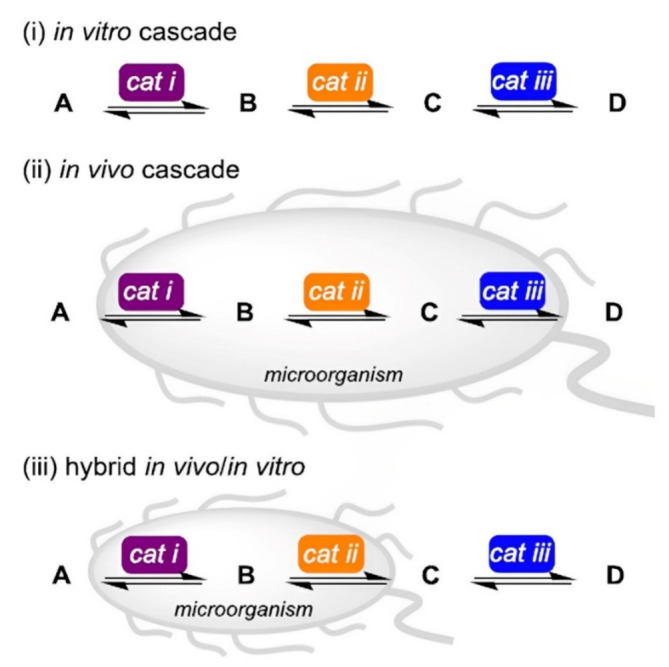
Comparison of (**i**) in vitro reaction and (**ii**) in vivo reaction with (**iii**) hybrid cascade reactions, utilizing potent and selective biocatalysts cat i–iii to develop product D from starting material A. Several parameters such as the availability of genetic sequences and recombinant enzymes, the need for cofactors, the absorption and release of substrates and products, and the metabolic stability influence which of these three possibilities is best for a given application [103], with permission from the American Chemical Society, copyright (2017).

As a fast-growing field, non-natural synthetic cascades enable the synthesis of complex useful compounds from simple precursors by combining consecutive biocatalytic events. Cascade reaction-based synthetic strategies are very helpful since they typically bypass several reaction work-up phases and the purification of all intermediary products [104]. Köhler et al. [105] reported on synthetic cascades that are enabled by combining biocatalyst with artificial metalloenzymes, as a homogeneous catalytic means to address synthetic challenges. The alternatives for biocatalytic retrosynthesis of a target molecule increase along with the toolbox of accessible biocatalysts, opening up new pathways including enzymatic conversions. Care must be taken while implementing such cascade reactions, especially when deciding whether to build the pathway in vitro or in vivo [103] (Figure 4). Biocatalysis has unquestionably advanced beyond one-step transformations to de novo cascade reactions which can be carried out in a “one-pot”, either with isolated catalysts or whole-cell systems and can even incorporate chemo-catalytic stages. Biocatalytic retrosynthetic design, which is expanding in breadth and applications as additional biocatalysts are researched and developed, allows for the anticipation of complexity cascades [106,107,108].

**Figure 5:**
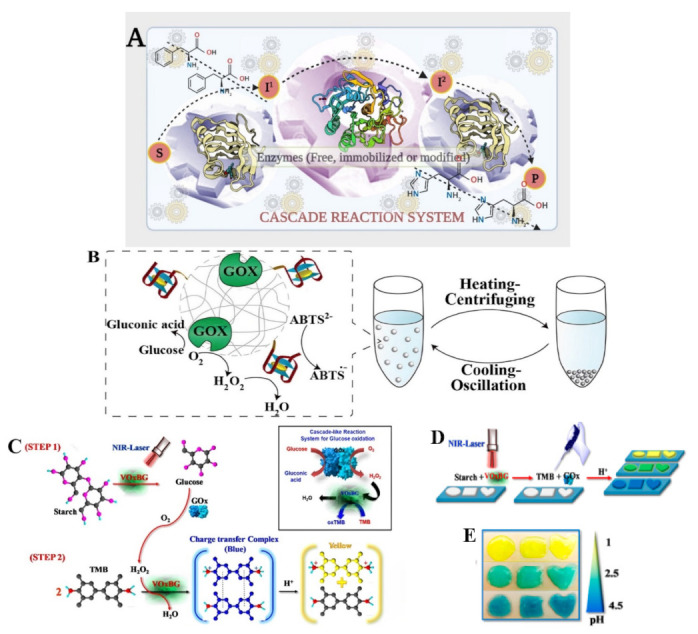
(**A**) Illustrating a general cascaded reaction mechanism and its operation. The figure made in Biorender.com. (**B**) A representation of the recycling use of the microgels through heating/centrifuging and cooling/oscillation cycles, as well as DNA/GOx cascaded catalyzed oxidation of glucose/ABTS^2-^. After the reaction, the microgels were removed from the reaction mixture using a heating–centrifuging technique and recycled by catalyzing a new DNA substrate following a cooling–oscillation dispersion procedure [46], with permission from Wiley periodicals Inc. (2018). (**C**) in the presence of an NIR laser, VOxBG catalyzes the conversion of starch into glucose (step I). In the presence of H_2_O_2_, GOx catalyzes the oxidation of glucose to produce H_2_O_2_, while VOxBG acting as HRP catalyzes the oxidation of TMB to produce colored compounds which changes in response to the different pH values (step II), (**D**) the photolithography process is illustrated, in a polyacrylamide hydrogel, starch and VOxBG are incorporated, (**E**) various geometric designs are created by photolithographing the hydrogel [125], with permission from the American Chemical Society, copyright (2020).

These synthetic cascades, which involve converting basic starting materials, such as frequent metabolic intermediates, into complicated target structures, are predicted to become more advanced as a result of access to a wider variety of various biocatalysts. Biocatalysts can now mediate a wide range of organic and chemical reactions, due to advances in multi-enzyme discovery and screening techniques as well as faster and less expensive gene synthesis [109,110,111,112,113]. The result is a continually expanding catalytic toolbox made up of naturally occurring or engineered enzymes [114,115,116], and synthetic enzymes [117,118] that can catalyze a wider variety of chemical transformations. Citrate synthase was tested using a label-free fluorescent enzyme assay based on CoA-Au(I) co-ordination polymers and applied in a multi-enzyme logic gate cascade. It was determined that using the TCA cycle’s cascade enzymatic interactions to process chemical synthesis revealed a simple and affordable logic system with faster detection [119]. Figure 5A shows a cascaded reaction mechanism and operation, Figure 5B shows a catalytic cascade reaction of glucose oxidation and heating-induced recycling of microgels, and Figure 5C,D indicate a hybrid multi-enzyme mimetic via a cascade reaction. According to Chauboon et al. [120], enabling effective flavin reduction and hydride transfer in a one-pot bioconversion of _L_-Arabinose to _L_-Ribulose in an enzymatic cascade is favorable toward a complete yield of the products. Sperl and Sieber [121] reviewed the status and recent advances of multi-enzyme cascade reactions and highlighted that employing artificial enzyme cascades for the integrated and other materials that can be controlled to synthesize a variety of desired chemicals regardless of the starting material available is the objective for the near future to sustainably improve life on earth. By eliminating the time-consuming isolation and reaction purification of intermediates, cascade reactions are unquestionably better than traditional step-by-step synthesis in terms of costs and waste. Additionally, higher yields can be obtained, and the atom economy is improved. Management and altering of adverse reaction equilibria as well as the potential manipulation of unstable intermediates are further advantages of cascade reactions [122,123]. The one-pot synthesis of (*R*)-α-Hydroxy ketones from meso- or racemic epoxides via enantioselective cascade biocatalysis revealed a green and high-yielding technique that could be useful for the preparation of certain chemical synthesis of which polymer materials could be efficient [124]. Thus, enzyme cascade systems are efficient and feasible approaches in the one-step synthesis of polymer, hybrid systems and other starter materials.

### 4.3. Advantages and Disadvantages of Cascade Reaction Systems

There is a widespread desire to combine several chemical processes into one reaction process. The difficulties of intermediate stability, isolation/purification of desirable chemicals, and solvent exchange are lessened if this is successful [126]. There are significant advantages in terms of space and resource utilization, as well as frequently in terms of process completion time [127]. Even though there are many excellent examples of chemical “one-step” synthesis that combine two or more processes, most of the time, incompatibility of conditions (particularly solvents, pH, and co-catalysts) necessitates performing reactions in sequence. Many of the difficulties in synthetic or semi-synthetic chemical reactions may be overcome by cascading reactions aided by several enzymes. Cascade enzyme reactions offer specific advantages. An additional reaction in a cascade reaction can stabilize partially unstable intermediates produced by enzyme reactions [128]. This has the specific benefit of making systems with reactive substrates easier to implement. By connecting with another process to steer the reaction in the desired direction, enzyme cascades provide the ability to accelerate reactions toward completion [103].

However, when developing new polymeric materials, it is necessary to take the drawbacks of enzyme cascades into account. Cascades may require multi-factorial optimization e.g., using a design of experiments approach [129], particularly where enzymes in the cascade have different cofactor requirements, to achieve the maximum activity. Enzymes are typically evolutionarily optimized for their biosynthetic pathways in vivo, rather than large-scale industrial application, which presents another hurdle for the inclusion of enzyme cascades into larger synthetic pipelines. Poor solvent compatibility with desired products [130], constrained substrate ranges [131], substrate flux sinks [132], and low turnover rates are some difficulties imposed by this challenge. However, to combat these limitations, it is now possible to synthesize a variety of enzymes in the quantities required by industrial applications due to advances in molecular biology techniques and the identification of new enzymes utilizing bioinformatics and added coupling chemical processes with biocatalysis for in vitro processes.

## 5. One-Step Synthesis for Enzyme Assemblies

### 5.1. Nano-Enzyme Assemblies

**Figure 6:**
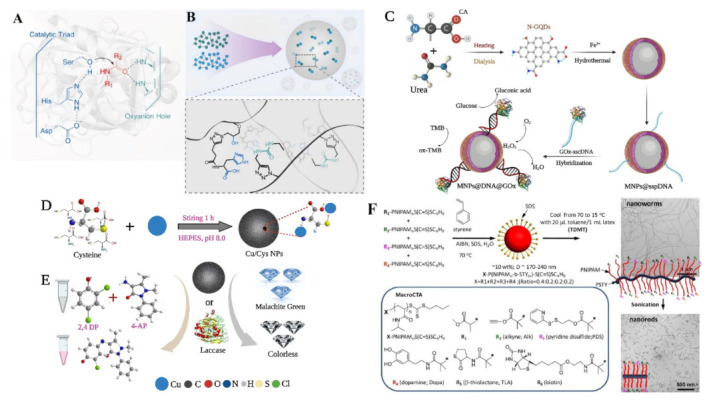
Schematic illustrations of the one-step nano-enzyme synthesis. (**A**) A hydrolytic enzyme with an oxyanion hole and a catalytic triad. In a Ser–His–Asp motif, the carboxyl group of aspartate forms a hydrogen bond with the imidazole domains of histidine. The hydroxy group of serine that is close by is subsequently deprotonated by the imidazole group to produce a nucleophile, (**B**) using nanoprecipitation in water to generate polystyrene-supported catalysts with urea and ACT groups. Here, a facile one-step method for synthesizing polystyrene-supported catalytic systems with integrated and tailored ACTs, urea groups, and hydrophobic environments is devised [136], with permission from Elsevier, copyright (2022), (**C**) fabrication of nanozyme-enzyme multi-catalyst system, reproduced from reference [137], (**D**,**E**) illustration of the synthesis of Cu/Cys nanozyme with laccase-like activity, reproduced from reference [134], (**F**) RAFT-Mediated emulsion polymerization in water followed by TDMT and ultrasonic cutting to generate multifunctional nanoworms and nanorods [138], with permission from the American Chemical Society copyright (2014).

Multi-enzyme catalysis requires enzyme assembly to improve catalytic efficiency and stability while progress in nanotechnology provides new materials and methods for the design of multi-enzyme cascade one-pot synthesis of the polymer [133]. Immobilization, self-assembly, and composite nano enzymes are the most used assembly methods. Figure 6 shows various nano enzyme assemblies employed in the one-step synthesis of polymer materials. By carefully combining copper ions and cysteine (Cys), Tang et al. [134] synthesized biomimetic copper–cysteine nanoparticles (Cu/CysNPs) with a stronger laccase-like activity than natural laccase (Figure 6D,E). The approach of immobilizing an enzyme on zinc sulfide–chitosan hybrid nanoparticles was discovered by Bahri et al. [135] and it considerably improved the materials’ thermal and storage stabilities, and resistance to pH extremes.

**Figure 7:**
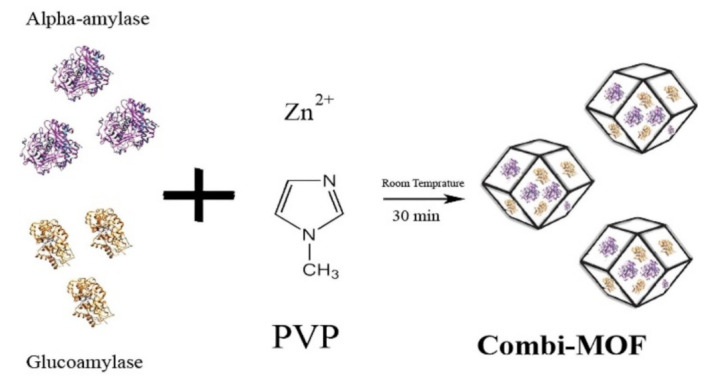
Fabrication of highly efficient combi-metal organic frameworks [139], adapted with permission from Elsevier, copyright (2018).

To enhance the stability of free enzymes in reactions, a variety of materials have been developed as support. Metal organic frameworks (MOFs) is one of the first support to be used and the efficient metal organic frameworks of α-amylase and glucoamylase were constructed, which could carry out multi-enzyme one-pot reaction in vitro, with higher catalytic efficiency and stability than the free enzymes [139]. Magnetic support that facilitates recycling has also been developed based on metal materials. For instance, Nobuaki et al. produced a complex made of layered titanate (TiOx), magnetic beads (MB), and horseradish peroxidase (HRP) and realized multiple recycling [140]. Figure 7 shows the fabrication of an enzyme–MOF combination.

**Figure 8:**
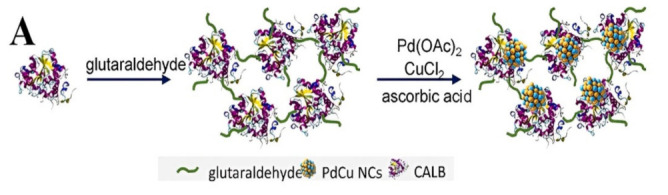

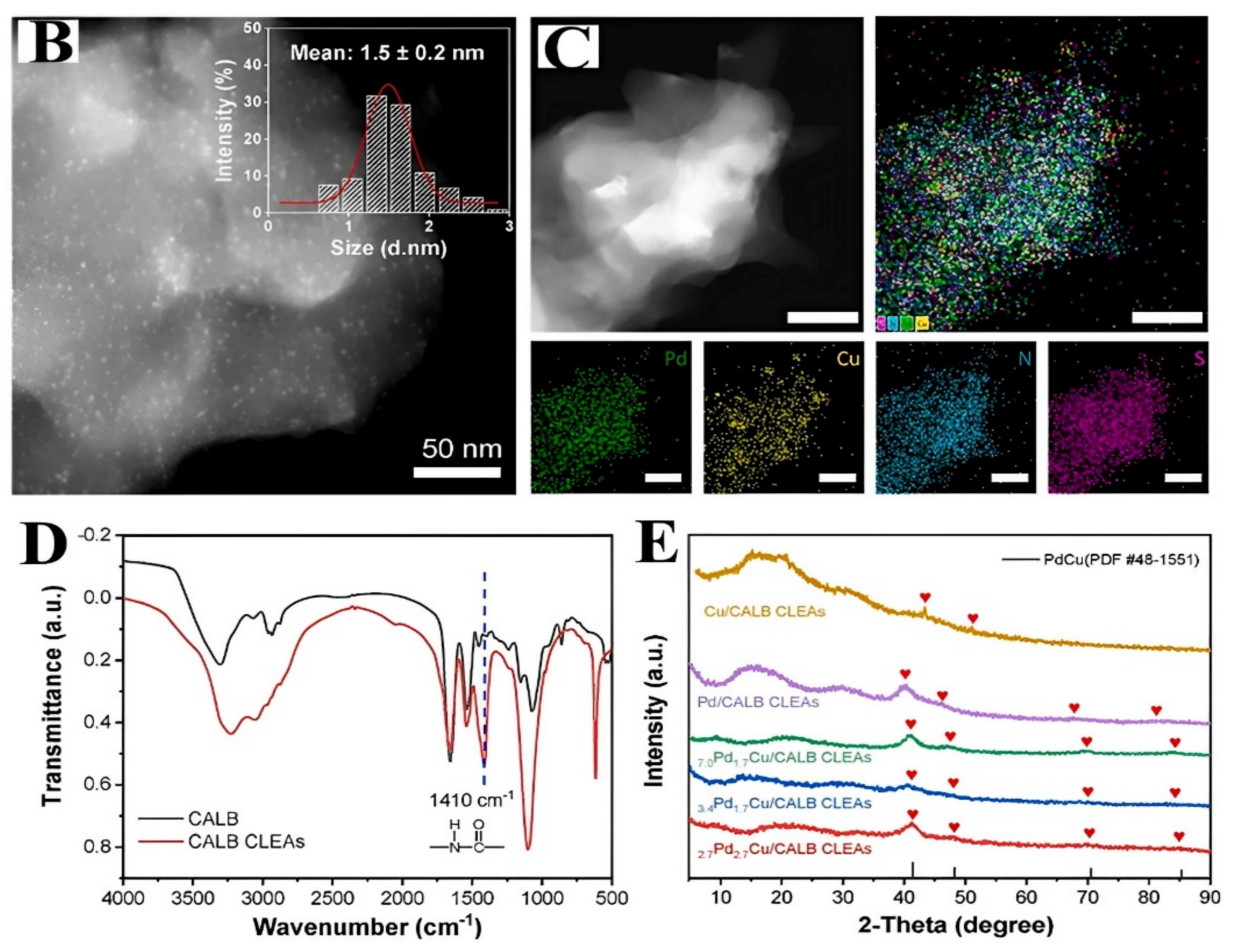
(**A**) Illustration of the of the in situ PdCu/CALB CLEA fabrication process. (**B**) HAADF-STEM picture of the hybrid catalyst composed of PdCu and CALB CLEAs. (**C**) An elemental map of Pd, Cu, N, and S that corresponds to (**D**) CALB and CALB CLEAs FT-IR spectra and (**E**) powder XRD patterns [141], with permission from Elsevier, copyright (2023).

In addition, catalytically active materials can also be used for the support itself. Shen et al. developed a flexible strategy for fabricating an effective multi-enzyme cascade system by immobilizing glucose oxidase (GOx) on ferriferous oxide nanocomposites functionalized with nitrogen-doped graphene quantum dots (Fe_3_O_4_@N-GQDs) using a DNA-directed immobilization [137]. The new enzyme immobilization method on graphene quantum dots provides a new idea for the design of enzyme catalysts. A recently created catalyst, 3.4Pd1.7 Cu/CALB CLEAs, has 2.6 times the activity of Pd/CALB CLEAs, which were previously employed extensively in the synthesis of (R)-N-[1-(4-(phenylethynyl)phenyl)ethyl] acetamide (Figure 8), verifying that an enzyme–bimetal complex catalyst is a powerful tool for the efficient and green asymmetric synthesis of high value organic compounds [141].

**Figure 9:**
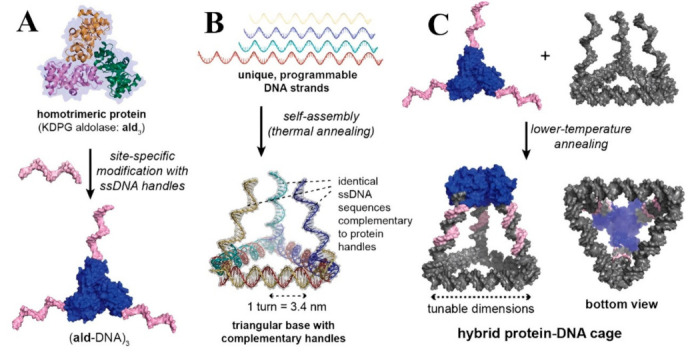
Hybrid DNA/protein cage architecture. (**A**) Addition of ssDNA handles to a site-specific reactive residue on an aldolase homotrimer (ald3) protein building block (**B**) self-assembly of three complementary handles and a triangular base for the protein from four distinct DNA strands and (**C**) by annealing the protein–DNA conjugate with a triangular base, a tetrahedral cage composed of both protein and DNA molecules is constructed. The cage’s proportions can be adjusted by altering the lengths of the DNA strands in the triangle-shaped base [147], with permission from the American Chemical Society, copyright (2019).

Non-metallic materials are also commonly used as support. Zhou et al. [142] reported a dual-enzyme cascade catalysis which is stabilized by immobilization in ZIF-8/GO composites obtained by GO-assisted co-growth. The low protein adsorption feature, biocompatibility, water solubility, good chemical resistance, and hydrophilicity of polymer material carriers are of concern. The surface functionalized nanofiber membranes were examined as a support material for enzyme immobilization, which increased the storage stability and reusability, and a novel amylase immobilized poly(vinyl alcohol) (PVA) nanofibers were developed [143]. Nanoparticle immobilization has contributed greatly to the biocatalysis of enzymes in different functional forms [144]. In addition to the immobilization of enzymes on substrates by various methods, the self-assembly of enzymes has also been developed, including assembly between different enzymes or with other catalytically active catalysts. This is particularly valuable if one or more enzymes are dimers/multimers, as it can promote enzyme ratios beyond 1:1, which can have benefits, such as bringing the catalytic activity closer to the desired ratio of enzyme activities [145]. For example, the simple one-step immobilization of zinc ions and adenine in self-assembling aqueous solution under mild conditions, while improving their activity and stability, enables multiple recycling, which will be helpful for the development of polymer synthesis catalysts [146]. Additionally, developing hybrid nanocages (Figure 9) made of self-assembling proteins and DNA will enable the development of nanomaterials that combine the benefits of both DNA and protein nanotechnology [147]. Wang et al. [148] achieved the array arrangement of the multi-reactions by alternately equidistant anchoring two model enzymes, glucose oxidase (GOx) and HRP, to the vertices of the rigid DNA tetrahedral unit so that the distance between adjacent cascades was adjusted to an optimal level to obtain high enzyme cascade catalytic efficiency.

**Figure 10:**
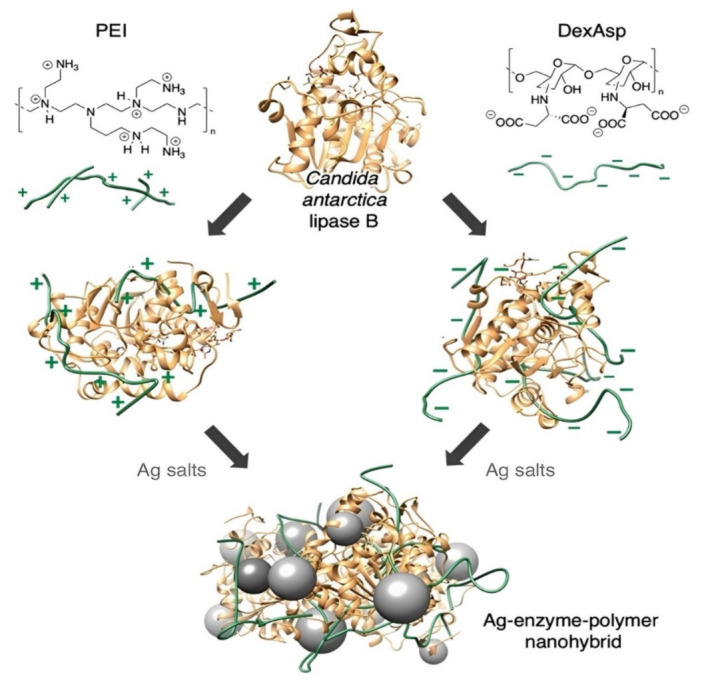
Synthesis of metal nano-biohybrids using enzyme–polymer conjugates. Illustrating the attempted prevention of mutual deactivation of the coupled enzyme and metal entities through poisoning effects in the nano-biohybrid synthesis by using polymer additives in the structure of the enzyme–metal nano-biohybrids [152], with permission from Wiley periodicals Inc., copyright (2022).

Due to the excellent properties of enzymes as catalysts, enzymatic polymer synthesis has always been an important direction in the synthesis of polymer materials. Based on the above enzyme assembly methods, researchers have developed several multi-enzyme cascade polymerization methods. In order to directly produce highly branched polyesters, Nguyen et al. [149] polymerized bio-based feed fractions such as tall oil fatty acid (TOFA), glycerol, azelaic acid, and pentaerythritol utilizing an immobilized *Candida antarctica* lipase B and the potential for enzymatic synthesis of alkyds. High value repeating disaccharide units make up the linear polysaccharide known as hyaluronan (HA). Li et al. [150] devised the sequential one-pot multi-enzyme (OPME) method for producing HA and its derivatives in vitro. A developed method for the production of lipophilic esters of hydroxytyrosol, which were synthesized from tyrosol and long-side chain carboxylic acids in a one-pot process by an enzymatic cascade of lipase M and tyrosinase supported by lignin nanoparticles [151]. Additionally, the synthesis of enantiomer-enriched O-heterocycles from alleles catalyzed by a cascade of bifunctional lipase metal bioheterozygotes was also achieved [152] (Figure 10). Thus, it can be inferred that enzymes assembled by various methods have great application prospects in polymer synthesis.

### 5.2. Self-Assembling Peptides

**Figure 11:**
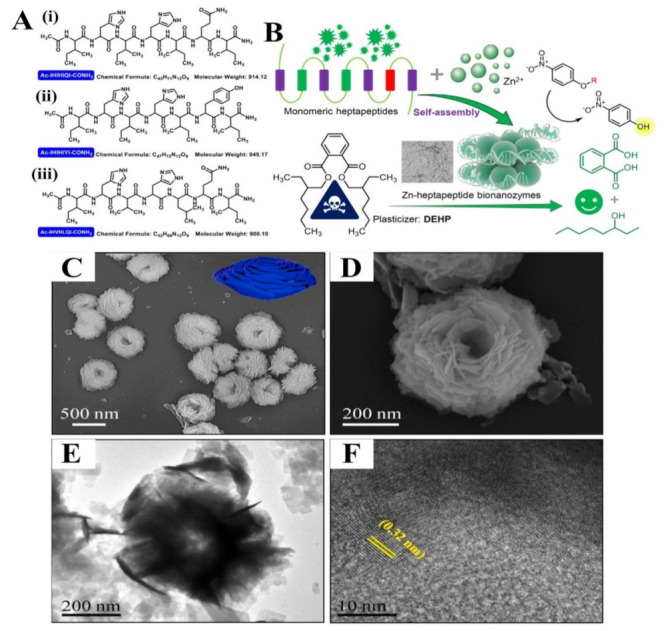
(**A**) Structural information of three heptapeptides ((**i**) Ac-IHIHIQI-CONH_2_, (**ii**) Ac-IHIHIYI-CONH_2_, (**iii**) Ac-IHVHLQI-CONH_2_)). (**B**) Schematic illustration showing the design of Zn-heptapeptide bionanozymes for hydrolysis of p-nitrophenyl esters and degradation of DEHP [153], with permission from Elsevier, copyright (2022). (**C**,**D**) SEM with difference magnifications, (**E**) TEM, and (**F**) HR-TEM images of BSA-Mn_3_(PO_4_)_2_⋅3H_2_O hybrid nanoflower (MnPNF) [154], with permission from Elsevier, copyright (2021).

Self-assembling peptides (SAPs) are ionic-complementary peptides that alternate hydrophilic and hydrophobic amino acid residues, with the advantages of their biocompatibility and ability to efficiently target molecular recognition sites which will be beneficial in one-step synthesis for effective catalysis of polymer materials. At last, SAPs play an important role, which could be used in encapsulation and catalysis. Liang et al. [153] created three oligopeptide bionanozymes with intrinsic hydrolase-like activity by achieving the zinc-induced self-assembly of histidine-rich heptapeptide, which shows great potential for environmental remediation. As for medical application, Dega et al. [154] used self-assembled bovine serum albumin-hydrated manganese phosphate nano-flowers (MnPNF) which works as a biomimic oxidase for GSH detection in human serum. Figure 11 shows the schematic information for designing SAPs. Self-assembling peptides also provide a new approach for the fabrication of integrated multi-enzymatic cascade systems that remains a great challenge. An arginine-rich peptide–Pt nanoparticle cluster (ARP-PtNC) nanozymes that mimic two typical enzymatic cascade systems of uricase/catalase and superoxide dismutase/catalase in natural peroxisome has been reported, which multiplied catalytic activity by introducing peptides [155]. As this section has been extensively reviewed elsewhere [156], it will not be discussed in detail here. Beyond this, it also confirmed that the rational design of peptide–nanozyme complex may provide a versatile and designed strategy to fabricate multi-enzymatic cascade systems, opening new avenues to researchers in this field.

## 6. One-Step Synthesis Application

### 6.1. Application in Polymeric Scaffolds

**Figure 12:**
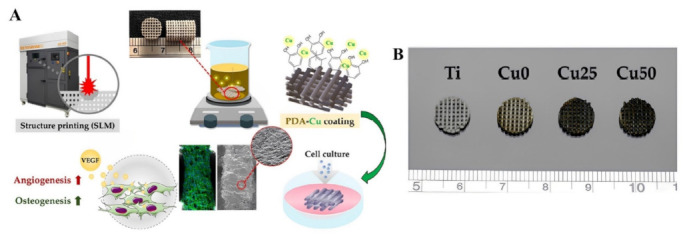
(**A**) Schematic representation of the Cu ion grafted by DA coating on the SLM-fabricated Ti6Al4V scaffold, which gave the scaffold the ability to stimulate angiogenesis and osteogenesis by releasing Cu ions. (**B**) Differential appearance of printed structures before and after PDA/Cu coating showing the changes in scaffold’s appearance [165], with permission from MDPI, Basel, copyright (2022).

Polymer materials have advanced over the past century, paving the way to potential industrial uses with the advancements in rising attention in the domains of polymer chemistry, nanotechnology, and biotechnology. Enzymes are powerful catalysts, but they are also very delicate molecules that can easily be denaturated beyond their normal environment. The application of one-step synthesis in producing polymer scaffolds has been reported in imidazole scaffolds [157], porous membrane-based scaffolds [158], multiple heterocyclic scaffolds [159], and the construction of β-Lactam scaffolds [160]. The successful fusion of enzymes with adaptable synthetic or natural polymers is made possible by a variety of opportunities provided by polymer chemistry, which can help resolve this problem. The design of functional, stable, and strong biocatalytic hybrid materials (hydrogels, nanoparticles, films, or capsules) has demonstrated effectiveness for a number of applications, including organic synthesis, bioremediation, biosensing, and biomedicine. Figure 12 shows the schematic preparation and printing of a polymeric scaffold for biomedical use. Immobilized enzymes have been employed in polymeric scaffolds [161,162,163,164,165]. Through the chemical modification of the protein’s sequence, by immobilizing the enzyme on stable supports, or by employing a combination of methods, the extensive application of enzymes in polymer scaffolds could be achieved [166]. Recently, the research and development of new scaffolds for tissue engineering have attracted a lot of attention. Particularly, the electrospun nanofibrillar scaffold materials are promising materials for use in biomedical applications [165,167,168] because they are produced from biocompatible and biodegradable polymers. Karahaliloğlu et al. employed an enzyme from the *Alcaligenes eutrophus* DSM 545 strain to produce cell-friendly bacterial electrospun PHB polymeric scaffolds. Through plasma polymerization modification, the nanofibrillar structures appeared to be effective substrates for cell attachment [169]. In a one-step surgical concept for cartilage and bone tissue engineering, the rapid attachment of resorbable polymeric scaffolds identifies both poly(_L_-lactide-*co*-caprolactone) and collagen type I/III as promising scaffold materials [170]. In the one-step synthesis of producing polymer scaffolds, the chain conformation of the polymer must also be considered because bulky and large polymers may obstruct the enzyme’s catalytic pocket and reduce its catalytic effectiveness. Overall, careful consideration of several characteristics is required for the design of an experiment that yields an enzyme–polymer hybrid that is catalytically active, reliable, and stable.

In tissue engineering, a scaffold serves as an important component as it functions as a three-dimensional structure for cell interactions, extracellular matrix production, and a transporter for delivering the right cytokines and growth factors to the repair site [171]. One advantage of polymeric scaffold for tissue repair is that it gives the newly formed tissue the appropriate structural and functional support that it needs. However, for scaffold tissue regeneration to be effective, it must be properly shaped on both the outside and inside to support cellular processes such as adhesion, proliferation, and differentiation, it must possess similar mechanical properties to those of the tissue repair site, it must be biocompatible, and must have the same degradation rate in the case of bone regeneration [172,173]. Tissue regeneration employs the use of synthetic polymers as they are biocompatible as well as easy to control in their three-dimensional structure and other surface features. Regardless, the downside to the use of polymeric scaffolds for tissue regeneration is that many scaffold properties need to be designed based on cell types for specific applications as scaffolds with increased surface roughness work best with osteoblast attachment while scaffolds with smooth surfaces work best with endothelial cells and fibroblasts [174].

The incorporation of polymer scaffolds in drug delivery systems has also been researched. A drug delivery system (DDS) involves any method that loads and delivers active compounds to target tissues to enhance the safety and effectiveness of drugs and the design of polymer implants or injectables by material researchers’ function in the repair of tissues as well as the cure of illness [175,176]. The use of these polymer injectables ensures minimal incision sizes and effective anatomical closing, and prevents the post-treatment step of removal surgery which is almost impossible with other thick or solid forms [177]. Additionally, polymer implants permit shape adaptation of the device which makes them advantageous compared to other scaffold-producing materials [178]. Polymeric scaffolds for DDS promote drug protection which in turn prevents side effects as implants are site specific and drug release and drug levels are controlled [179,180]. Biocompatible scaffolds are also used in the delivery of proteins, cells, and genes and these applications improve their capacity for the designs of novel treatments for diseases such as tumors and pathogen-derived microbials, and as a tool for tissue repair in regenerative medicine to stimulate biological responses [175].

### 6.2. Challenges of the Application of One-Step Synthesis in Scaffolds

Despite their obvious benefits and widespread use, polymer scaffolds generated through one-step synthesis have certain practical drawbacks, including a lack of mechanical strength for load-bearing applications and a failure to scale up. However, different research teams have proposed several strategies to surpass the constraints listed above. In tissue engineering, it has some practical drawbacks, including poor cellular penetration and ingrowth, the toxicity of chemical residues in electrospun fibers or postprocessing, insufficient mechanical strength for load-bearing applications, and a sluggish manufacturing rate [181]. Some scaffold manufacturing strategies could also account for these challenges. For example, the freeze-drying technique has the disadvantage of high energy consumption, the development of uneven hole diameters, and the use of cytotoxic solvents to dissolve the polymers [182]. In addition, the solvent casting method and particulate leaching are time consuming, require extensive time to evaporate the toxic compounds, have limited membrane thickness, and have mechanical properties [183]. Additional challenges such as tissue architecture, simultaneous seeding, and vascularization have also been reported and reviewed extensively [184]. Thus, more hybrid structures and the incorporation of enzyme composites are advocated for the construction of scaffolds to lessen these associated limitations.

## 7. Conclusions and Prospects

One-step synthesis is an efficient and simple approach for the construction of polymeric or organic/inorganic hybrid functional materials, and there is a need to carefully adjust them to suit industrial standards for high productivity to potentially replace the expensive and environmentally harmful advanced organic chemistry techniques. There have been only modest industrial successes despite scientific advancements and exploratory efforts to fully understand one-step synthesis and its functional dimensions for polymeric materials. The current review focused on the significant factors among the numerous determining factors for optimal enzyme functioning in the synthesis and production of polymer materials. By altering the synthesis interaction, the optimal enzyme design and the ideal catalyst choice can both influence the polymer function and product profile. However, the enzyme–polymer synthesis scientific community still requires a friendly standardized methodology, biological and chemical engineering, as well as enhanced protein model-based methodologies to expedite the structural unification designs for the synthesis of enzyme-catalyzed polymers and/or other materials.

Besides the free, immobilized, or modified enzymes, several non-enzymatic one-step methods also exist that might have a significant effect on the synthesis of the polymer-coupled complexes. Although this paper is focused on the entire synthesis of enzyme polymers, some of the most recent breakthroughs in one-step organic/inorganic chemical synthesis were discussed as prospective directions to be modified and merged in enzyme systems. Green chemistry has successfully used enzyme-mediated polymer synthesis at low cost and under mild reaction conditions. In addition to the various polymerization reactions, existing polymers can also undergo modification reactions to develop new polymeric materials. However, compared to enzymatic polymerization, enzymatic modification has produced far fewer polymers so far. Enzymatic catalysis has been utilized for the synthesis of polymer conjugates, selectively functionalized polymers, the cross-linking of polymers, the modification of the surface characteristics of polymers, and other chemical modifications.

In addition to the added advantages for enzymes in the one-step process, there are some limitations to their use which include (1) enzyme catalysts have fewer types of substrates and reactions than do chemical catalysts; (2) the availability of enzymes and their cost; (3) although there is substantial turnover, responses seem to be slow. In many instances, the amount of catalyst is the issue, normally due to the catalytic active site contained in a single enzyme molecule. However, there is still opportunity for development given these drawbacks in terms of the large reaction rate and extremely high selectivity, a low amount of by-products, enzymes being renewable, and immobilized enzymes can be readily recovered and recycled, functional in organic and supercritical fluids, are renewable resources, and in most cases, products are biodegradable.

It is desired that using the one-step approach to perform green polymer chemistry can enable the establishment of a sustainable society in the future. Enzymes offer several prospects to expand our reach to a wide variety of polymers with roots in chemical and biosynthetic pathways due to their extensive selectivity and preference for the available substrate pool. We conclude by stating that generating materials via enzyme-wise synthesis of polymer partners can comprehensively shape the future generation of enzyme–polymer catalysis research.

## Figures and Tables

**Table 1:** Overview of the use of some enzymes in the one-step/pot synthesis of polymer materials.

Enzymes	Approach	Product	Application	Refs.
Lipase	One pot	Fe_3_O_4_/PPy	Biocatalyst for industrial applications Utilized in the building of a wide range of immobilized enzymes for laboratory and industrial purposes	[52]
Lysozyme, lipase, glucose oxidase and horseradish peroxidase	One pot	Ca-DDVA and Zn-DDVA	Can be used to carry out enzyme delivery/release for nutrition or biomedical applications	[53]
Tyrosinase	One pot	EGCG-chitosan hydrogel	Broad tissue regeneration applications that require immune modulation	[54]
*Candida Antarctica* lipase B (CALB)-Novozym 435	One pot	Optically active polymer (*r*-polymer)	It can be applied for large-scale manufacture of optically active polymers	[55]
Lysozyme	One pot	Graphite oxide and calcium-carboxylate based Metal-Organic Frameworks (GO-CaBDC)	Biocatalysis under acidic conditions	[56]
Glucose oxidase	One pot	Chitosan-reduced graphene oxide nanoparticles (CS-RGO-AuNPs)	Applicable in the construction of biosensors	[57]
Tris-(benzyltriazolylmethyl) amine (TBTA)	One-pot	Polyvinylbenzyl chloride	Used to develop marine coatings with antifouling properties Preparation of coating materials that absorbs naturally abundant Cu (II) and Cu(I) ions from seawater.	[58]
Peroxidase	One pot	Lignin-composite (SC_2_/SC_6_-CafAc-L_1_, SC_2_/SC_6_-CafAc-L_2_)	Synthesis of artificial lignin-based composites.	[59]
Lysozyme (Lyz)	One pot	Poly (AHA-co-PMA-co-EDMA) monolithic	Utilized in the separation of polycyclic aromatic hydrocarbons, nucleosides and nucleobases, alkaloids, peptides and proteins	[60]
Glucose oxidase/catalase	One step	Hybrid interpenetrating polymer network (HIPN) hydrogel	Applicable for enzyme immobilization	[61]
Glucose oxidase and alcohol oxidase	One step	Fluoropolymer (Nafion) and noble metal palladium (Pd-NPs	Tuning properties of polymer membranes Construct enzymes reactions with optimized sequences such as improved mechanical and operational stability and reduced enzymes and polymer leakages.	[62]
*Candida Antarctica* lipase B (CALB)-Novozym 435	One step	Unsaturated oligoesters and polyesters	Utilized in the applications for unsaturated polyester resins or photosensitive coatings.	[63]
*Candida Antarctica* lipase B (CALB)-Novozym 435	One step	Poly(*p*-dioxanone-*co*-butylene-*co*-succinate) copolyesters	Potential to be utilized in biomedical application	[64]
β-Glucosidase and horseradish peroxidase (HRP)	One step	Poly(2-(β-glucosyloxy) ethyl acrylate) (PGEA), poly(2-(β-glucosyloxy) ethyl methacrylate) (PGEMA), and poly(4-(β-glucosyloxy) butyl acrylate) (PGBA)	Biomedical applications	[65]
Horseradish peroxidase (HRP)/glucose oxidase (GOD)	One step	Dendritic polyglycerol (dPG)	Applicable in medicine related bio interfaces	[66]
Horseradish peroxidase (HRP)/glucose oxidase	One step	Molecularly imprinted polymers	Specific recognition and facile immobilization of enzymes in complex extract samples.	[67]
Lysozyme	One step	Polystyrene	Biomedicine, nongenetic engineering of cells, environmental protection, energy operations, and catalysis	[68]
Glucose oxidase, peroxidase	One step	Porphyrin functionalized CeO_2_ nanoparticles (Por-CeO_2_ NPs)	Potential application in biotechnology and clinical diagnosis as peroxidase mimetic	[69]
β-galactosidase, glucose oxidase, and horseradish peroxidase	One step	Hydrogels	Production of advanced microfluidic systems for serial and/or parallelized catalytic enzymatic reactions	[70]
*Candida Antarctica* lipase B (CALB)-Novozym 435	One step	Poly e-caprolactone (e-CL) and poly e-thiocaprolactone (e-TCL)	Synthesis of new biomaterials for different green and biomedical applications	[71]
Levansucrase and Endo-inulinase	One step	Fructooligosaccharides and oligolevans	Applied in the food industry	[72]
Peroxidase-like nanozyme	One pot		Visual assay for tyrosine activity	[73]

## Data Availability

Not applicable.

## Abbreviations and Acronyms

ABTS	2,2′-Azino-bis(3-ethylbenzothiazoline-6-sulfonic acid) diammonium salt
ACTs	artificial catalytic traid system
AHA	6-azidohexanoic acid
APD	ambient pressure drying
AuNPs	gold nanoparticles
CaBDC	calcium-carboxylate based metal organic frameworks
CafAc	caffeic acid
co-PMA	co-propargyl methacrylate
co-EDMA	co-ethylene dimethacrylate
CALB	*Candida antarctica* Lipase B
CS-RGO	chitosan-reduced graphene oxide
CLEAs	cross-linked enzyme aggregates
dPG	dendritic polyglycerol
DEHP	degrading di(2-ethylhexyl) phthalate
e-CL	e-caprolactone
e-TCL	e-thiocaprolactone
EGCG	epigallocatechin gallate
GOD	glucose oxidase
GO	graphite oxide
HIPEs	high internal phase emulsions
HIPN	hybrid interpenetrating polymer network
HRP	horseradish peroxidase
LNA	local nucleic acid
Lyz	lysozyme
MOFs	metal organic frameworks
NBR	nitrile butadiene rubber
NIR	near infrared spectroscopy
NPs	nano particles
OPME	one-pot multi-enzyme
PCM	phase change materials
Pd	palladium
PEG	polyethylene glycol
PEG-g-PDMAEMA	poly(ethylene- glycol)-graft-poly(N,N-dimethylaminoethyl methacrylate)
PGBA	Poly(4-(β-glucosyloxy) butyl acrylate
PGEA	Poly(2-(β-glucosyloxy) ethyl acrylate
PGEMA	Poly(2-(β-glucosyloxy) ethyl methacrylate
pNIPAM	Poly-*N*-isopropylacrylamide
PVA	polyvinyl alcohol
RAFT	reversible addition-fragmentation chain transfer
SPD	supercritical drying
TBTA	tris-(benzyltriazolylmethyl) amine
TDMT	temperature directed morphology transformation
TEM	Transmission electron microscope
TMB	3,5,3′,5′-tetrame- thylbenzidine
TOFA	tall oil fatty acids
UCST	upper critical solution temperature
VOxBG	hexacyanoferrate Berlin green analogue complex
Zn-DDVA	Zinc-5,5′-dehydrodivanillate
ZIF-8	zeolitic imidazole framework-8
